# Anterior Displacement of a Posterior Malleolar Fragment Through the Syndesmosis: A Case Report

**DOI:** 10.7759/cureus.42451

**Published:** 2023-07-25

**Authors:** Don Koh, Kinjal Mehta

**Affiliations:** 1 Orthopaedics, Changi General Hospital, Singapore, SGP

**Keywords:** orthopaedic surgery, orthopaedics trauma, xray, foot and ankle fracture, ankle and foot

## Abstract

Ankles are the most common site of injury in lower limb fractures. Despite this, the classification of the Maisonneuve fracture is still highly controversial, perhaps due to its low incidence. Typically described as a proximal fibular fracture with associated injury to the syndesmosis and medial structures secondary to an external rotation mechanism, the injury often necessitates surgical intervention to restore joint stability for good functional outcomes. A 32-year-old lady sustained a pronation external rotation injury resulting in a proximal fibula fracture with disruption of the distal tibiofibular syndesmosis as well as an associated posterior malleolar fracture with displacement of the fragment anteriorly through the syndesmosis to the ventral aspect of the ankle joint. The patient underwent surgical fixation of the posterior malleolar fracture as well as repair of the syndesmosis with a screw. This report aims to highlight the details of a Maisonneuve fracture with the rarer associated posterior malleolar fracture, and its anterior displacement through the syndesmosis, as well as provide a narrative review of the current literature.

## Introduction

Rotational fractures represent the majority of ankle fractures [1]. The Maisonneuve fracture was first described in 1840 [2], and refers to a combination of a fracture of the proximal fibula along with a disruption of the distal tibiofibular syndesmosis. The accepted mechanism of Maisonneuve fracture is pronation external rotation according to the Lauge-Hansen classification [3]. Maisonneuve fractures account for only 5% of all ankle fractures treated by surgery [4]; therefore, there is only limited data about them. The classification of these fractures remains controversial, with various authors describing differing characteristics of the fracture, such as injury mechanism, morphology, and position.

A retrospective study by He et al. reported a posterior malleolar as the involved structure in a Maisonneuve fracture in the majority of the patients (82.93%) [5]. This case report aims to highlight the interesting displacement pattern of the posterior malleolar fracture fragment through the disrupted syndesmosis, as well as provide a review of the current literature on Maisonneuve fractures.

## Case presentation

A 32-year-old female with no past medical history was brought in by ambulance after sustaining a fall whilst riding her kick scooter. She reported losing her balance and sticking out her left foot onto the pavement in an attempt to brake. The mechanism of injury was reported to be a pronation external rotation and resulted in the patient sustaining an open fracture of her left ankle.

Upon physical examination, the patient was noted to have a gross deformity of the left ankle joint with associated diffuse swelling, as well as a small (< 1 cm) puncture wound over the lateral medial malleolus. There was significant tenderness over the posterior aspect of the lateral malleolus on palpation as well as a limited range of motion of the ankle joint due to pain. Examination of the fibula revealed no tenderness on palpation along its length. The distal neurovascular status of the foot was intact, as well as no pain on palpation along the fibula. The patient also sustained a left elbow fracture-dislocation, which was initially reduced in the ED and subsequently managed conservatively (the details of which are not covered in this report).

**Figure 1:**
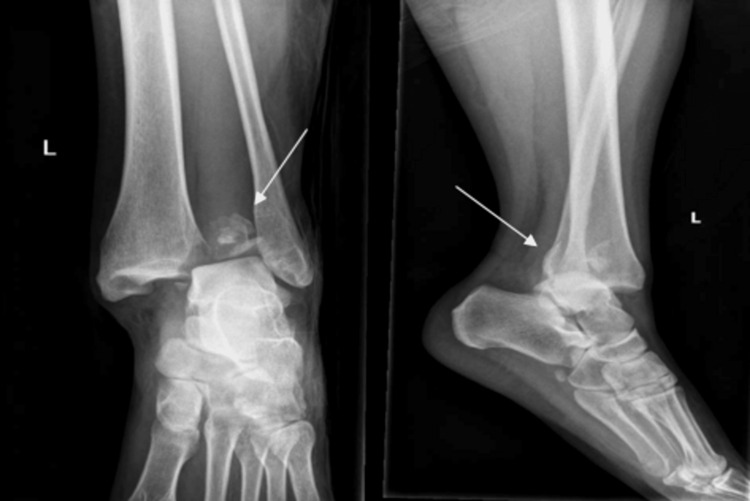
Left ankle anterioposterior/lateral X-ray images depicting a posterior malleolar fracture with displacement of the fragment into the syndesmotic gap

Initial plain film radiographs of the affected limb were performed as protocol. X-rays of the left ankle revealed a posterior malleolar fracture with displacement of the fragment into the anterior segment of the ankle through the widened syndesmosis (Figure 1).

**Figure 2:**
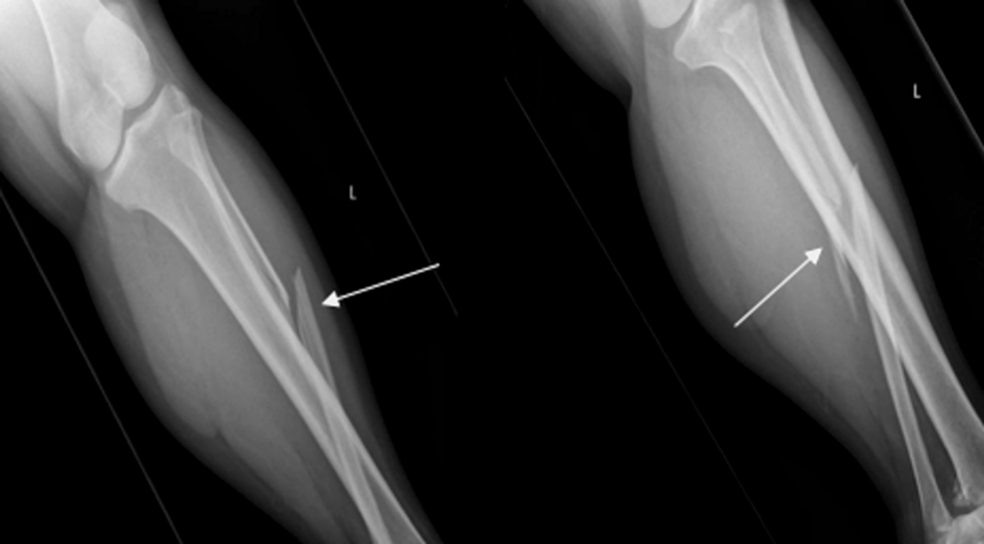
Left tibia/fibula anterioposterior/lateral X-ray images depicting a fracture of the proximal third of fibula

X-rays of the tibia and fibula also showed a slightly displaced oblique fracture at the proximal one-third of the fibula (Figure 2).

Initial management was that for an open fracture in view of the wound puncture wound over the fracture site, and included irrigation of gross contaminants of the wound and administration of intravenous antibiotics as per guidelines published by the British Association of Plastic, Reconstruction and Aesthetic surgeons (BAPRAS) and British Orthopaedic Association (BOA).

The patient was brought into the operating theatre for wound debridement and manipulation and reduction (M&R). Intra-operative findings confirmed a left open posterior malleolar fracture with syndesmotic injury, graded as a Gustilo-Anderson grade 2 in view of the skin wound measuring less than a centimetre, and only mild periosteal stripping of the exposed fragment [6]. The syndesmosis was widened, and the posterior malleolar fragment was estimated to be roughly 30% of the ankle joint surface area. There was no significant soft tissue loss or damage to any nearby structures, specifically the great saphenous nerve and the posterior tibial neurovascular bundle. Following wound exploration, debridement, and subsequent irrigation, the fracture was reduced with the aid of intra-operative imaging.

**Figure 3:**
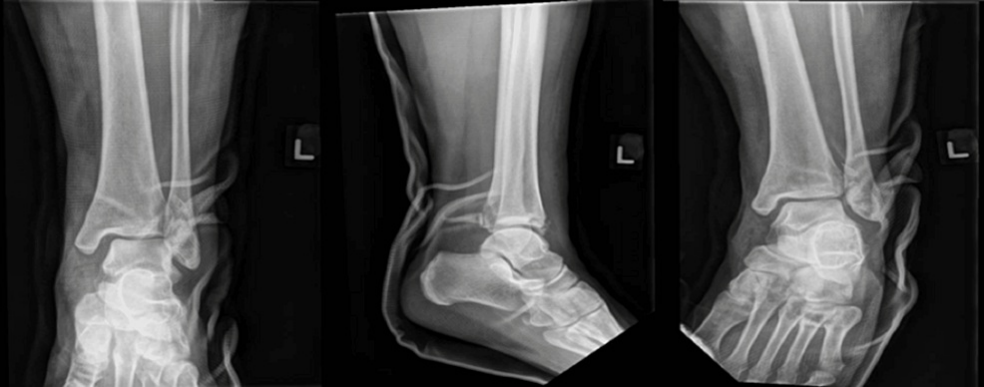
Post manipulation and reduction left ankle mortise/lateral/anterioposterior X-ray images

Post manipulation and reduction, X-rays revealed satisfactory relocation of the ankle joint (Figure 3).

**Figure 4:**
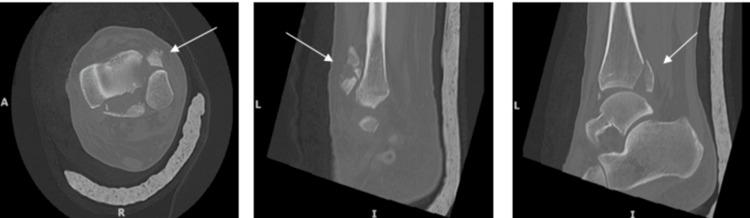
Computerized tomography left ankle axial (Left) and sagittal (Middle and Right) cut images showing the displaced fragment and the posterior malleolar fracture

Computerized tomography (CT) scans were subsequently performed for operative planning. CT images revealed the posterior malleolar fragment to be anterior to the fibula, likely being displaced anterolaterally through the widened syndesmosis during prior M&R (Figure 4).

**Figure 5:**
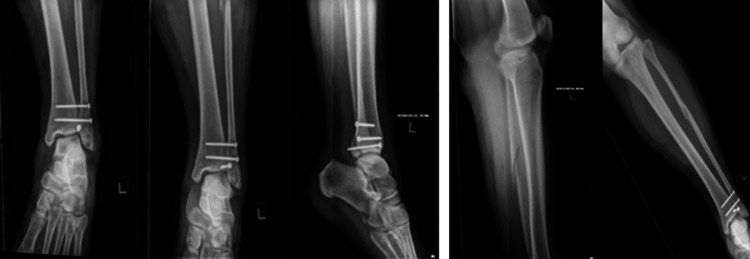
Post-operative left ankle anterioposterior/lateral/mortise views (Left), and left tibia/fibula anterioposterior/lateral views (Right)

Definitive fixation was performed via an anterolateral approach with an incision made over the syndesmosis. Dissection was performed with the superficial peroneal nerve visualized and protected down to the fragment, which was displaced to the anterolateral aspect of the syndesmosis. The fragment was placed posteriorly and the posterior malleolar reduced and held with a Kirschner wire before placing a partially threaded cancellous screw to compress the fracture via lag technique to achieve absolute stability for the articular fracture. The syndesmosis was then reduced and two 3.5 mm cortical screws were inserted. Post-operative X-rays are shown in Figure 5.

**Figure 6:**
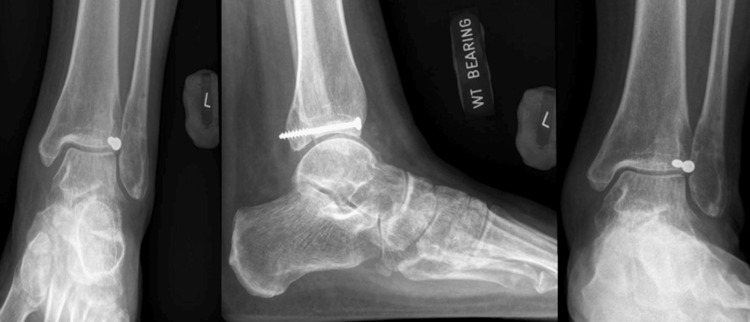
Final left ankle X-ray anterioposterior/lateral/mortise views

The patient was kept non-weight bearing for a total of eight weeks post-operatively. The patient had an uneventful recovery and also underwent the removal of the syndesmotic screw at the eight-week mark. At the six-month follow-up, the patient was walking and reported good functional outcomes with scores of 70 (out of 100) for the Olerud Molander score, and 73% for the Foot and Ankle Outcome Score. Ankle X-rays at the time of the final review were noted to have satisfactory healing of the fracture with a preserved mortise (Figure 6).

## Discussion

Ankles are the most common anatomical site of injury in the lower limb, accounting for 22.6% of all lower limb fractures [7]. On the initial presentation of ankle injuries, care must be taken to adequately evaluate the proximal fibula, especially when the reported mechanism of injury is that of pronation external rotation. A case series of five patients with Maisonneuve fractures who were only diagnosed after repeat clinical consults up to 17 weeks by Taweel et al. highlights how easily the Maisonneuve fracture can be missed [8], likely owing to the fact that the clinical and radiographical examination is usually directed to the main complaint of pain over the ankle joint.

Biomechanical studies have shown that the disruption of the distal tibiofibular syndesmosis results in loss of congruency of the ankle mortise, and hence the stability [9] and normal motion of the ankle joint. Surgical intervention to restore the anatomical relationship has been shown to be critical in achieving good functional outcomes [10]. Screw fixation is still the choice of surgical fixation by most surgeons for syndesmotic injury. Patients treated with the suture-button device were shown to have better functional outcomes based on the American Orthopaedic Foot and Ankle Society (AOFAS) score compared to those treated with syndesmotic screws (91.06 and 87.78, respectively), as well as having reduced rates of complications such as implant removal, failure, and malreduction [11]. However, a survey of 100 surgeons in 2021 by Shafiq et al. showed an overwhelming majority (97%) still favoring screw fixation [12], perhaps due to the technique still being relatively new compared to the conventional syndesmotic screw, along with the lack of exposure of its technique to surgeons and lacking data of long-term outcomes.

The fracture pattern seen in our case report was of the posterolateral oblique type, in keeping with the findings in the study by He et al., who also found it to be the most common pattern in posterior malleolar fractures as part of a Maisonneuve fracture [5]. Although having the most common fracture pattern, there has been no known case of a displacement pattern of the fragment anteriorly through the syndesmosis as seen in our case. The posterior malleolus is known to prevent posterior talus subluxation by contributing to tibiotalar load transfer [13]. Statistically significant differences in outcomes in patients with ankle fractures with an associated posterior malleolar which was fixed compared to those which were unfixed based on the Manchester-Oxford Foot questionnaire (MOXFQ) score (24.03 and 20.10, respectively) were shown in a study by Jeyaseelan et al. [14]. Surgical fixation for all posterior malleolar fractures should therefore be considered to restore ankle stability and better functional outcomes, as well as prevent complications such as secondary osteoarthritis of the joint [15].

## Conclusions

To the author’s best knowledge, there have been no similar reports of fracture displacement patterns such as that described in this case. Due to a widened syndesmosis, fragments could be displaced anteriorly or posteriorly through the gap. Repair of the syndesmosis remains crucial in achieving good functional outcomes.
